# Digital pathology and artificial intelligence in renal cell carcinoma focusing on feature extraction: a literature review

**DOI:** 10.3389/fonc.2025.1516264

**Published:** 2025-01-24

**Authors:** Ming-Yue Li, Yu Pan, Yang Lv, He Ma, Ping-Li Sun, Hong-Wen Gao

**Affiliations:** ^1^ Department of Pathology, The Second Hospital of Jilin University, Changchun, Jilin, China; ^2^ Department of Urology, The Second Hospital of Jilin University, Changchun, Jilin, China; ^3^ Department of Orthopedics, The Second Hospital of Jilin University, Changchun, Jilin, China; ^4^ Department of Anesthesiology, The Second Hospital of Jilin University, Changchun, Jilin, China

**Keywords:** digital pathology, artificial intelligence, deep learning, WSI, RCC, prediction, diagnosis, prognosis

## Abstract

The integrated application of artificial intelligence (AI) and digital pathology (DP) technology has opened new avenues for advancements in oncology and molecular pathology. Consequently, studies in renal cell carcinoma (RCC) have emerged, highlighting potential in histological subtype classification, molecular aberration identification, and outcome prediction by extracting high-throughput features. However, reviews of these studies are still rare. To address this gap, we conducted a thorough literature review on DP and AI applications in RCC through database searches. Notably, we found that AI models based on deep learning achieved area under the curve (AUC) of over 0.93 in subtype classification, 0.89-0.96 in grading of clear cell RCC, 0.70-0,89 in molecular prediction, and over 0.78 in survival prediction. This review finally discussed the current state of researches and potential future directions.

## Introduction

### Renal cell carcinoma

Renal cell carcinoma (RCC) refers to a diverse group of malignant tumors originating from the epithelial cells of the renal tubules, with an increasing incidence (1). The 2022 Fifth World Health Organization (WHO) Blue Book divided RCC into morphologically and molecularly defined RCCs, classifying RCCs into three major subtypes: clear cell RCC (ccRCC), papillary RCC (pRCC), and chromophobe RCC (chRCC), which together constitute over 90% of RCC cases (2). CcRCC is the most common subtype (70%-80% of cases), which is graded using the Fuhrman classification, now replaced by the World Health Organization/International Society of Urology (WHO/ISUP) grading system (3). In addition to histological classifications, the Fifth WHO update introduced molecular-driven categorizations (4), emphasizing the importance of molecular profiling for RCC diagnosis and prognosis.

### Digital pathology, WSI, and pathomics

Digital pathology (DP) is a technique that utilizes digital technology and computer-aided tools to convert images from traditional glass slides into high-resolution digital formats (5). Initially, DP focused on digital image acquisition. With the advancements in artificial intelligence (AI), DP has now integrated AI to further enhance its capabilities. This integration enables advanced data storage and retrieval, while offering automated image analysis and diagnostic support (5).

Whole-slide imaging (WSI) is a significant technology in DP that utilizes automated microscopes to capture high-resolution detailed images (6). It employs computer algorithms for precise stitching and processing, enabling the quantification of features such as shape, size, and color in pathological images (6). A randomized controlled trial compared six pathologists’ diagnostic accuracy using glass slides versus WSIs for the same cases. The results showed no significant differences, confirming diagnostic equivalence (7).

Pathomics is an emerging subject, which is the integration of pathology and omics, aiming to utilize computational techniques to process and interpret pathology image data (8). Through high-throughput feature extraction, visualization, and quantification, it aims to develop predictive models that uncover potential biomarkers (9).

### Artificial intelligence

In the medical milieu, AI employs advanced algorithms like machine learning (ML) and deep learning (DL), to analyze complex medical data, assist diagnosis and treatment planning, and optimize patient outcomes (9, 10). It has been integrated with diagnostic technologies such as X-rays, CT scans, magnetic resonance imaging, ultrasounds, and gene sequencing, achieving satisfactory results in predicting diagnosis, prognosis, and treatment response (9).

In pathology, the application of AI covers many aspects, such as cytological screening, morphological quantification analysis, tissue pathology diagnosis, and prognosis assessment (11, 12). M. Giulietti et al. (13) compared the prediction performance of AI-based and non-AI-based predictors, with the former performing slightly better. A. Distante et al. (14) assessed the precision, efficiency, and objectivity of histopathological analysis, suggesting that AI can overcome intra and interobserver variability and time consumption.

One of the bottlenecks in the application of WSI integrated with AI is interpretability, also known as black box features (15). The features automatically extracted by the initial AI model during training are often high dimensional and abstract. Although these features are effective in optimizing model performance, they lack biological significance, making it difficult to explain the relationship between these features and prediction tasks (15). In response to the growing demand for interpretability in medical fields, researchers primarily focus on improving the steps of feature extraction and prediction models to enhance this aspect (16). For example, Zhang et al. (17) proposed a Structural Priors Guided Network (SPG-Net) that not only achieves high segmentation accuracy but also incorporates prior structural knowledge, making the model’s predictions more interpretable.

The previous reviews summarized the applications of AI and DP in the diagnosis and prediction of pathology and RCC (18, 19). However, they did not focus on the extracted features. This study aimed to address this gap by providing a comprehensive overview of methods for enhancing the interpretability of AI and mitigating its black box nature in future research. We focused on summarizing and analyzing the existing literature related to feature extraction techniques in AI for RCC, offering insights into how these methods can improve model interpretability.

## Materials and methods

A literature search was performed on the PubMed and the Web of Science database platforms in January 2024. The search was carried out using the following terms: “((renal cell carcinoma) AND ((artificial intelligence) OR (machine learning) OR (deep learning))).” In addition, a manual search was performed to identify additional potentially relevant articles.

English publications were included if published between 2017 and January 2024, due to advancements in AI, particularly DL and CNNs, which began impacting DP and improving model accuracy. Nonpeer reviewed articles, case reports, comments, and conference summaries were excluded. A duplicate search was performed in Endnote software (version 20), together with the manual screening of titles and abstracts. Literature screening and evaluation were conducted following the PRISMA2020 checklist (20).

## Results and discussion

**Figure 1:**
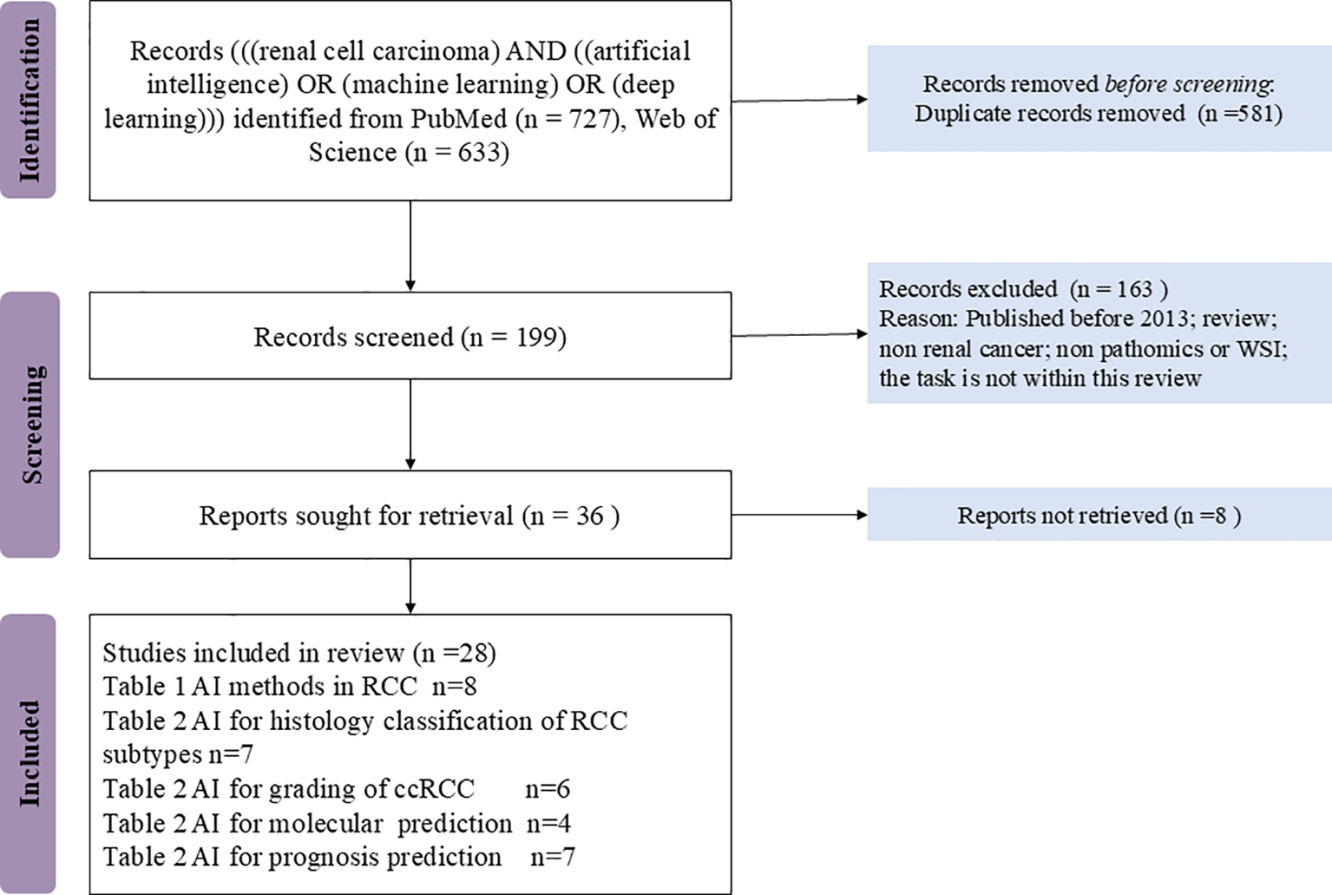
Flow chart of studies included via databases according to the PRISMA strategy.

**Figure 1** provided the flow diagram for the PRISMA strategy. In all, 1032 records were excluded, and the 28 remaining full texts were checked for suitability.

The text mining software programs SATI (http://sationline.cn/) and Voyant (https://voyanttools.org/) were used to read and analyze full records and cited references. Of the 28 records in the study, 2 were not in the Web of Science core collection, and 26 records were included in the text mining analysis. The results of the analyses of the keywords, authors, institutions, and citations were shown in **Supplementary Materials 1**–**4**.

Although the results based on text mining were only objective index statistics, and did not involve the overall scientific evaluation of the literature, they can help us to explore the rich information of research in this field.

### AI algorithms in RCC

The current AI approaches used for classification and prediction include supervised, weakly supervised, self-supervised, and unsupervised learning (21). Supervised learning involves fully labeled data, which provides broader context and clearer relationships within the data, potentially enhancing the model’s ability to explain its predictions. However, its scalability is limited by the time-consuming, labor-intensive, and error-prone labeling process (21). In weakly supervised learning, image-level (WSI-level) labels are commonly used, which offer coarse annotations for entire images. These methods typically provide less detailed contextual information compared to fully supervised methods, which use pixel-level labels or bounding boxes (22). Unsupervised learning methods depend solely on the intrinsic properties of the data, such as similarity, consistency, or relationships, to uncover underlying structures or representations, often applied in medical image analysis Meanwhile, self-supervised learning, which falls between supervised and unsupervised approaches, leverages unlabeled data by creating pretext tasks that help the model learn useful representations without explicit labels (23). A summary of AI algorithms in RCC is provided in **Table 1**.

**Table 1:** AI methods in RCC.

Ref	Aims and Tasks	Key AI Technologies	Features and Interpretability
Lu et al. (22)	Classify RCC subtypes	Weakly supervised	CLAM heatmaps of identifying morphological features
Faust et al. (23)	Analyze the clinical and biologic relevance of the intra and interpatient subgroups	Unsupervised	512dimensional deep learning feature vectors Feature activation map (FAM)
Chen et al. (25)	Predict prognosis	Supervised	Nuclei segmentation: nuclear atypia, abundant tumor cellularity, and other features. Both CAMs and gradient based attribution techniques
Lee et al. (27)	Predict prognosis	Semi-supervised	Pathological context features and interpretability through the attention score: 1) local features: small clear tumor cells, pleomorphic tumor cells or tumor cells with rhabdoid differentiation, tumor cells with clear to eosinophilic cytoplasm, glomeruli or glomeruli tubules; 2) surrounding local features: cystic changes and stromal hyalinization, solid alveolar or papillary growth and stromal hemorrhage, necrosis and lymphocytic infiltration
Chen et al. (28)	To search and retrieve WSIs with similar morphological features from large repositories	Self-supervised	The patches contain meaningful ROIs, and they can be visualized to provide model interpretability
DiPalma et al. (29)	Classify RCC	Self-supervised	Gradient weighted Class Activation Mapping (Grad CAM) visualizations
Chen et al. (30)	To improve patient risk stratification	Supervised	Morphological features, including nuclear size, nuclear morphology, mitoses, and cell concentration; molecular features. Interpretability: attention and gradient based interpretability; a custom visualization tool that overlays attention weights
Schulz et al. (32)	Predict prognosis	Supervised	Histopathologic features (tumor vasculature, hemorrhage, and necrosis); cell nuclear division, nucleoli, nuclear morphology, nuclear size, perinuclear area. A sliding window approach to visualize unimodal classification WSIs; CAMs

### Supervised learning

In supervised learning, one of the most widely used deep learning (DL) architectures is the Convolutional Neural Network (CNN), which consists of multiple layers of convolutional filters that can automatically extract features from images (24). CNNs have demonstrated superior performance in image segmentation, detection, and generation tasks (24). However, these methods also require either manual annotation of gigapixel WSIs or large datasets of WSIs with slide level labels (25). The input to a CNN is a small patch image obtained from segmenting the WSI, focusing on local features, such as morphological changes or different growth patterns of tumor cells, but often failing to pay attention to contextual background features (25). For example, Chen et al. (25) proposed Supervised Multimodal Fusion, an interpretable strategy for end-to-end multimodal fusion of histology images and genomic features. This approach not only utilizes CNNs but also incorporates other methods, such as: 1) graph CNNs for learning cell graph features, 2) Graph Convolutional Networks (GCNs) for survival outcome prediction from histology, and 3) the Kronecker product of gated feature representations along with a gating-based attention mechanism.

### Weakly supervised learning

In weakly supervised learning, the researchers implemented Graph Neural Network (GNN) to extract these features using attention and integrated gradients (IG) for model interpretation to fully exploit the contextual features of WSI (26). However, these methods overlook the spatial interactions of local pathological features and lack interpretability for contextual features in WSI (26). To address these limitations, Lee et al. (27) proposed a semi-supervised tumor environment related context learning using graph deep learning (TEA graph), which is a GNN based method that represents WSI through super patches and can analyze the context of spatial interactions of histopathological features. Additionally, in study (22), a weakly supervised clustering-constrained attention-based multiple instance learning (CLAM) method was introduced. CLAM utilizes attention-based learning to identify subregions of high diagnostic value while applying instance-level clustering to refine and constrain the feature space, eliminating the need for pixel-level annotations, ROI extraction, or sampling.

### Unsupervised learning

Several approaches have been proposed in both unsupervised and self-supervised learning. For instance, in study (23), an unsupervised pretrained CNN was used as a feature extractor for histopathology, generating all groupings through unsupervised learning techniques such as dimensionality reduction and clustering. In study (28), a self-supervised image search method for histology pipelines was introduced, utilizing a Vector Quantized Variational Autoencoder (VQVAE) trained on a large dataset to improve feature extraction. Additionally, in study (29), a self-supervised deep learning method, known as Resolution-Based Distillation, was proposed. This technique distills the learned representation knowledge from a teacher model into a student model trained at a lower resolution, while minimizing the impact on classification performance.

Both weakly supervised and unsupervised methods face greater challenges in interpretability compared to supervised algorithms that use more comprehensive annotations (21). These challenges include obtaining reliable and robust weak labels, addressing ambiguities and uncertainties in the data, and evaluating and interpreting results (21). Nonetheless, there is growing interest in developing weakly supervised and unsupervised learning methods for medical image analysis, as they can leverage rich unlabeled or partially labeled data, thereby reducing the annotation burden.

### Model fusion techniques

Model fusion techniques are commonly used to deal with multimodal data, including pathomics, radiomics, genomics, and proteomics data and other clinical medical record data, as these diverse data sources provide complementary information that can enhance performance in prediction tasks (30). Model fusion can be performed at different stages of an algorithm pipeline, in early fusion, late fusion, or hybrid fusion (21, 31). Model fusion can be achieved through ensemble learning, or multimodal deep learning methods, which use neural networks to identify joint representations from different data modalities (28). For example, in study (30), a supervised multimodal fusion DL model was designed to integrate whole-slide images and molecular profile data using a weakly supervised multimodal deep learning algorithm. Schulz et al. (32) developed a supervised multimodal model that can integrate multiple medical images (WSI, CT, MRI) and genomic data and fuses the multi-source output information through the attention layer. Chen et al. (28) proposed an image search method based on self-supervised learning to optimize the histological analysis process. They enhanced the interpretability of the model by visualizing the ROIs, providing an intuitive tool for pathologists to evaluate the basis of model decision-making.

### Application of AI in RCC

**Figure 2:**
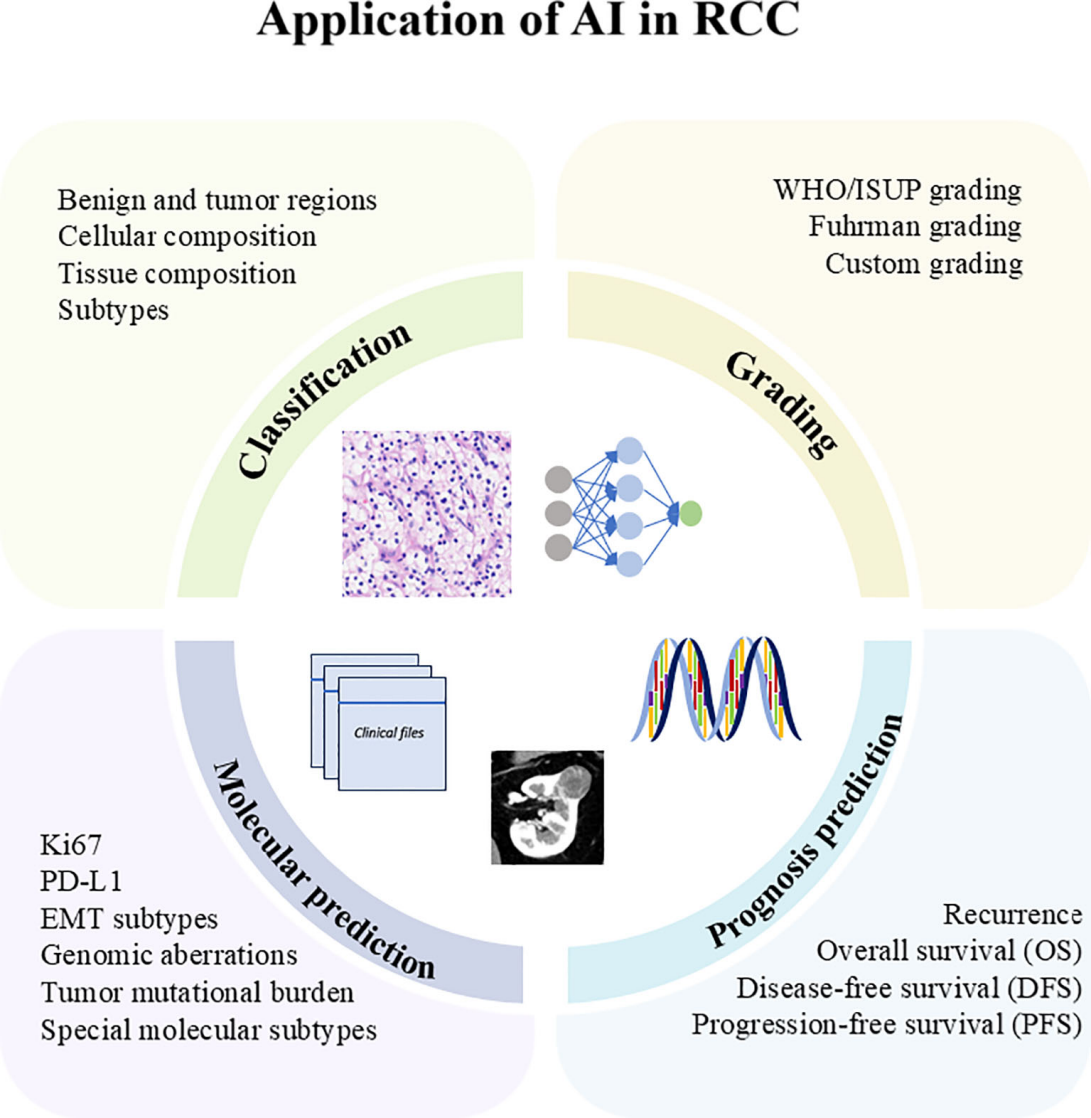
Application of AI in RCC.

The application of AI in RCC has enhanced classification, grading, molecular prediction, and prognosis prediction, as shown in **Figure 2**. In the following sections, we summarized and elaborated on these applications in **Table 2**.

**Table 2:** AI for histology classification of RCC subtypes.

Ref	Aims and Tasks	Key AI Technologies	Features and Interpretability	Evaluation on Validation set
AI for classification of RCC				
Fenstermaker et al. (33)	Classify ccRCC, pRCC, chRCC	CNN	ROIs; no features mentioned	Accuracy 0.979
Abdeltawab et al. (34)	Classify fat, renal parenchyma, clear cell RCC, and clear cell papillary RCC	Pyramidal model: three CNNs;	ROIs; no features mentioned	Accuracy 0.957
Zhu et al. (35)	Classify ccRCC, pRCC, chRCC, renal oncocytoma, and normal tissue	CNNs: ResNet 18	ROIs; no features mentioned; GradCAM	AUC 0.98 Mean F1score 0.92
Tabibu et al. (36)	Classify ccRCC, pRCC, chRCC and normal tissue	Pretrained Resnet 18 and Resnet 34, DAGSVM (data imbalance)	Tumor morphology features, cell nucleus features, and other relevant characteristics from the histopathology images (to predict the survival outcome)	Accuracy 0.941
Marostica et al. (37)	Classify the benign regions, chRCC, ccRCC, and pRCC	Transfer learning on VGG16, Inceptionv3, and ResNet50	Histopathology image regions containing malignant cells (identifying image patches with malignant cells)	AUC of the best model 0.953
Ohe et al. (38)	Classify clear and eosinophilic phenotypes	Transfer learning on DCNN	Cytoplasm; WHO/ISUP grade, vascularity based architectural category, tumor related necrosis, three tier morphologic immunophenotypes: desert, noninflamed; excluded, peritumoral immune infiltration; and inflamed, intratumoral immune infiltration	AUC 0.929
Chen et al. (39)	Classify ccRCC and normal renal tissues	LASSO	The shapes, sizes, textures, pixel intensity distributions and proximity relations of the primary and secondary objects	AUC 0.970
AI for grading of ccRCC				
Fenstermaker et al. (33)	Fuhrman grading	CNN	ROIs; no features mentioned	Accuracy 0.984
Kruk et al. (40)	Fuhrman grading	ML; SVM classifiers:an ensemble of classifiers; feature selection method: FM; GA; RF; CFS; FCBF	Numerical descriptors of nuclei:texture, morphometry, color and histogram descriptions	Accuracy 0.904
Tian et al. (41)	Classify ccRCC into either low or high grade	ML; classification: Ensemble; Lasso regression; Elastic net; Ridge; Linear SVM; RF	72 nuclei 2D histological features: nine morphological features, 15 intensity-based features, and 48 texture-based features	Extended test set AUCROC 0.96 Accuracy 0.89
Holdbrook et al. (42)	Grading: low grade (Fuhrman grade 1 and 2) or high grade (Fuhrman grade 3 and 4)	ML; Patch Classification: SVMs, logistic regression and AdaBoost; Image Classification: SVM	The location of prominent nucleoli: 1) histogram of polar gradient, 2) enhanced histogram of polar gradient 3) exclusive component analysis feature and 4) raw pixel intensity values	FV score: correlation with an existing multigene assay–based scoring system:(R = 0.59)
Chanchal et al. (43)	Grading	RCCGNet: a CNN block called shared channel residual block	Visualization of nuclear morphology, nucleolar prominence, and nuclear membrane irregularities; GradCAM	F1score 0.8906 Accuracy 0.9014
Khoshdeli et al. (44)	Classified six categories: normal, fat, blood, stroma, low grade granular tumor, and high grade clear	CNN: a shallow and a deep model (GoogLeNet)	Not explicitly mentioned	F1score GoogLeNet 0.99 Shallow CNN 0.92
AI for molecular prediction of RCC				
Marostica et al. (37)	Predict genomic aberrations: KRAS CAN, WT1, EGFR, VHL, et al.	Multimodal DCNN to integrate WSIs, multiomics, and clinical data	Histopathology image regions containing malignant cells; cell morphology	AUPR greater than 0.7
Chen et al. (46)	Classify the EMT subtypes (Mes and Epi: related genes by hierarchical clustering)	Transfer learning on Inception v3; SGD optimization	Epi subtype: a looser arrangement, big cell gap, nucleoli absent or inconspicuous, pink granular eosinophilic cytoplasm Mes subtype: densely packed, surrounded by arborizing vasculature, the large multinucleate cells with empty cytoplasm, abundant immune infiltration CAM for visualization	AUC 0.84, accuracy 0.749, specificity 0.722, sensitivity0.753
Acosta et al. (47)	BAP1, PBRM1, and SETD2 in ccRCC	DL, VGG19	Nuclear Feature: 36 features quantifying aspects of nuclear size, shape, color, and texture; area of nuclear channel intensity; Haralick feature etc.	BAP1 AUC 0.77-0.84
Cheng et al. (48)	TFE3 Xp11.2 translocation in RCC	ML models (logistic regression, SVM with linear kernel, SVM with Gaussian kernel, and RF)	Nucleus and image level feature: 52 differential image features (related to the size and roundness of nuclei)	AUC 0.886
AI for prognosis prediction of RCC				
Tabibu et al. (36)	Predict survival outcome of ccRCC	ML: LassoCox model	Tumor regions detected by the CNN: 13 tumor shape features and 6 nuclei shape features	Association between combined image features and survival outcome (P < 0.01)
Marostica et al. (37)	Predict OS	CNNs coupled with multitask logistic regression	GradCAM visualization	Distinguish longer term survivors from shorter term survivors (logrank test P = 0.02)
Wessels et al. (50)	Predict 5yOS in ccRCC	Multivariable logistic regression; univariable Cox regression; CNNs	Features in the CNN prediction model: the morphology of the nucleus and nucleolus, accompanied by inflammatory reactions	In univariable Cox regression, the CNN prediction model showed a hazard ratio of 3.69 (95%CI: 2.60–5.23, P < 0.01)
Gui et al. (51)	Predict ccRCC recurrence	DL based WSIs analysis	Manually annotate the tissue regions and map the tumor area on WSIs	AUC at 3, 5, and 7 years of OS prediction: 0·787; 0·780; 0·823
Cheng et al. (52)	Predict prognosis	ML: a lasso regularized Cox proportional hazards model	150 patient level features; ten types of cell level features: characterizing nucleus size, shape, texture, and distance to neighbors; lengths of the major and minor axes of cell nucleus and the ratio of major axis length to minor axis length, mean pixel values	Lasso Cox risk index was an independent prognostic factor (P = 2.31e^-4^, hazard ratio = 2.26); the survival curves stratified by the lasso Cox risk index (logrank test P = 0.014)
Cheng et al. (53)	Predict the prognosis of pRCC	ML: lasso regularized Cox regression	Topological features in renal tumor microenvironment; morphological and intensity features	AUC of 0.78
Chen et al. (54)	Predict DFS	ML-based pathomics signature (MLPS)	Nucleus parameters and intensity parameters such as Nucleus Circularity, Nucleus Min caliper, Nucleus Hematoxylin OD mean, Nucleus Hematoxylin OD min and Cell Eosin OD	The AUC at 1, 3, 5, and 10 years of DFS prediction: 0.895, 0.90, 0.885 and 0.859

### AI for classification

Supervised CNN architectures and techniques are commonly employed for the classification of RCC (33–36). The working pipeline begins with pathologists manually annotating regions of interest (ROIs) on the pixel level in WSIs. These ROIs are then segmented into reasonably sized patches for classification at the patch level. During this process, data augmentation techniques are applied to preprocess the WSIs and address the issue of small datasets (33, 35).

For example, in study (35), the model not only achieves whole-slide classification but also visualizes key indicative regions and features on the slides, thereby enhancing its explainability. To further improve interpretability, the Gradient-weighted Class Activation Mapping (Grad-CAM) method is utilized with (AUC) values of 0.97 (95% CI: 0.96–0.98) for external set. E. Marostica et al. (37) integrated VGG16, Inceptionv3, and ResNet50 with multiomics and clinical data, and extracted features like cell morphology to diagnose the RCC subtypes, predict patients’ survival outcomes, Malignant cells are divided into ROI to identify malignant cells and complete the above tasks. However, the specific features extracted were not explicitly mentioned in the subsequent task of linking histopathological images with genomic data to reveal molecular morphology. Y. Yasukochi, et al. (38) employed a deep convolutional neural network (DCNN) trained via transfer learning on WSIs to predict the eosinophilic phenotype of RCC in a high AI score group. They extracted features related to clear and eosinophilic phenotypes and angiogenesis gene signatures. The independent validation set achieved an AUC of 0.929 for predicting clear or mixed/eosinophilic phenotypes, though external validation results were not provided. Chen et al. (39) extracted a total of 346 useful quantitative image features, including shapes, sizes, textures, pixel intensity distributions, and proximity relations in WSI for diagnosis of ccRCC patients. The AUC value of the model was 0.970 in the test cohort, and 0.814 in the external validation cohort.

### AI for grading of ccRCC

The classic WHO/ISUP grading system for ccRCC relies on the morphological characteristics of the nucleus, in particular on the presence of nucleoli at different magnifications (3). Key steps in the structured pipeline of automated grading systems based on ML technology include identification of ROIs, segmentation of nuclei, numerical descriptors pertaining to nuclei features, feature selection, and classification (40–42). Most studies utilize fivefold or tenfold cross validation techniques and image enhancement strategies to reduce the impact of small datasets (42).

For instance (**Table 2**), studies (40–42), have extracted cellular features such as cellular information included texture, morphometry, color, intensity, histogram descriptions and pleomorphic features. When building an automated classification system, selecting and improving classifiers is a crucial step, and some studies have proposed improved classifiers based on traditional SVM, RF, such as ensemble learners (40). In studies (33, 43, 44), classic architectures of DL, such as ResNet, Inception, and DL algorithms improved based on classic architectures are also used for the grading task. Chanchal et al. (43) proposed a novel shared channel residual (SCR) block to share the information between different layers and strengthens the local semantic features. Satisfactory accuracy and F1 scores were obtained by pathologists marking the tumor region ROI and extracting nuclear morphology visualization, nucleolar protrusion and nuclear membrane irregularities.

### AI for molecular prediction

Molecular driven categorizations of RCC, such as TFE3/TFEB rearranged RCC, and ALK rearranged RCC, have been added into the Fifth WHO, due to the importance of molecular profiling in RCC tumorigenesis, prevalence rates, diagnosis and prognosis (2). However, traditional morphological diagnosis based on microscopic pathological sections often falls short in accurately identifying tumor molecular heterogeneity. Additionally, gene expression analysis is not readily implemented in routine clinical practice due to cost and technical challenges (45). In response, AI technology utilizes DL algorithms to explore the correlation between morphological features and molecular characteristics extracted from tumor tissue images, consistently inferring molecular tumor subtypes from conventional histology.

For example (**Table 2**), Q. Chen et al. (46) developed an EMT gene signature that was used to classify ccRCC into epithelial and mesenchymal subtypes through DCNN, but the sample size used in that study was relatively small, and the neural network was trained on a single data set. Acosta et al. (47) developed a DL model to identify mutations in BAP1, PBRM1, and SETD2, achieving commendable performance. Cheng et al. (48) designed differential image features that are closely related to the size and roundness of the nucleus. Through in-depth study and analysis of these features, a variety of ML models have shown good diagnostic performance in distinguishing TFE3 Xp11.2 translocation-associated RCC from other types of RCC.

The use of AI technology to analyze pathological sections, extract features, and predict molecular subtypes offers pathologists a new perspective (47). However, this field is still in its early stages, and more data and in-depth studies are needed to validate the accuracy and reliability of AI’s association with RCC molecular characteristics.

### AI for prognosis prediction

Accurate prognostic predictions are crucial for clinical decision-making and evaluating therapeutic effects in patients with renal cell carcinoma (RCC), leading researchers to focus on developing clinical risk models (49). In prognostic prediction tasks, regression models in machine learning, such as Cox regression, Lasso-regularized Cox regression, and logistic regression (which is also employed for classification tasks), are commonly utilized (36). The typical workflow involves first extracting relevant features from WSIs, followed by using machine learning classifiers for risk scoring and classification (sometimes for feature transformation or further analysis rather than direct classification). Finally, survival outcomes are predicted using regression models (36, 50).

For example, F. Wessels et al. (50) established a univariate logistic regression model based on CNN prediction model and a multivariate logistic regression model combining CNNs prediction and clinicopathological parameters (we only showed univariate logistic regression results in **Table 2**). The results in the validation group showed that the AUROC of both models was 0.88. Gui et al. (51) combined the WSI score with a score based on six single nucleotide polymorphisms (SNPs) and the Leibovich score based on clinical pathological risk factors, to construct a multimodal recurrence scoring system. They found that the multimodal recurrence scoring system had higher predictive accuracy than single modality scores and clinical pathological risk factors, and could more accurately predict the recurrence free interval (RFI) for localized renal cell carcinoma patients. Cheng et al. (52) extracted nucleus features and topological features in the renal tumor microenvironment and eigengenes from functional genomics data to predict ccRCC prognosis. This team conducted another study (53) to predict patient prognosis in pRCC by extracting topological features included the histogram of cooccurrence of nucleus patterns and bag of edge histogram (BOEH) features spatial arrangement of different cell patterns in the tumor microenvironment. The author mentioned that the prediction effect of the integrated model is better than that of the model considering a single factor (52) and the proposed topological features were superior to traditional clinical features and cell morphology and intensity features in predicting patient outcomes (53). The group led by Shanghai Jiao Tong University School of Medicine (54) used QuPath digital pathology software (55) (a commonly used digital pathological analysis software) to carry out cell and nuclear segmentation and detected pathological signatures as machine learning based pathomics signature (MLPS) to predict the clinical outcomes of ccRCC patients. There are a total of 43 different pathological features, mainly nuclear and intensity parameters, used in the MLPS classification system, and satisfactory survival prediction based on MLPS classification has been obtained.

## Conclusions and future prospects

This article summarized relevant literatures on the application of AI and DP in RCC and outlined the methods currently employed to enhance interpretability, which is especially critical in the medical field. Visualization techniques are often used to visually highlight meaningful features and regions recognizable by pathologists to increase interpretability (23, 43). Features extracted based on WSI include but are not limited to color, texture, shape features, topology, etc. Tumor cell morphological (36, 37), and tumor microenvironment features (25, 27, 39) are common in RCC researches. The prediction tasks based on the above extracted features can achieve satisfactory results, while studies that do not provide extracted features often need to increase credibility by improving the interpretability of the algorithm (33, 36).

Although great progress has been made in the application of AI technology to the medical field, many challenges remain. A conflict is seen between the limitations of computing resources, the extent of original research materials, and the requirements for computing power and size of the data set for the use of robust AI models (56). Therefore, improving computing power and upgrading scanning equipment are issues that computer engineering should continue to address. Although the use of large databases has made up somewhat for the problem of insufficient data set in a single research center, the following issues still exist. 1. Extracting meaningful information from huge databases is still an arduous task. 2. The curse of dimensionality for high-throughput sequencing data leads to serious overfitting problems. 3. The quality of these data cannot be fully guaranteed, and the lack of multicenter external validation affects research reliability. The application of AI to the medical field also involves ethical issues, the optimizing of workflow, acceptance by pathologists, providing real assistance clinically, and delineating responsibilities for the resolution of clinical problems that need to be addressed.

Targeted therapy, chemotherapy, and immunotherapy are important adjuvants to surgical intervention in RCC (57). Therefore, the diagnosis and prognosis prediction of RCC based on clinical data, imaging data, pathological characteristics, and genes information are worth studying further. However, based on the findings of this review, researches on RCC primarily focus on three major histological subtypes, effectively identifying molecular aberrations and accurately predicting prognosis remain key challenges for future studies. Given the rarity of cases with specific molecular subtypes, the necessity for multicenter and collaborative studies is particularly pronounced. Furthermore, the integration of multimodal data from pathology, radiomics, genomics, and proteomics (58), along with the application of advanced AI technologies such as Large Language Models (59) is expected to make significant contributions to this field.

Our study has some limitations. The number of collected literature is limited, and only English articles and reviews on Web of Science and PubMed database are included, without articles of other languages or types. Our follow up plan is to develop systematic research to find more scientific and unified valuable feature extraction methods to break through the bottleneck of interpretability.

## Data Availability

The data sets used and/or analyzed during the current study available from the corresponding author on reasonable request.

## References

[1] Siegel RL Miller KD Jemal A . Cancer statistics, 2016. CA Cancer J Clin. (2016) 66:7–30. doi: 10.3322/caac.21332 26742998

[2] Moch H Amin MB Berney DM Comperat EM Gill AJ Hartmann A . The 2022 world health organization classification of tumours of the urinary system and male genital organs-part A: renal, penile, and testicular tumours. Eur Urol. (2022) 82:458–68. doi: 10.1016/j.eururo.2022.06.016 35853783

[3] Delahunt B Cheville JC Martignoni G Humphrey PA Magi-Galluzzi C McKenney J . The International Society of Urological Pathology (ISUP) grading system for renal cell carcinoma and other prognostic parameters. Am J Surg Pathol. (2013) 37:1490–504. doi: 10.1097/PAS.0b013e318299f0fb 24025520

[4] Smith SC Wobker SE . The new WHO 2022 category of molecularly defined renal carcinomas: Accessible to practicing pathologists. Am J Clin Pathol. (2023) 160(6):545–8. doi: 10.1093/ajcp/aqad101 37589571

[5] Jahn SW Plass M Moinfar F . Digital pathology: advantages, limitations and emerging perspectives. J Clin Med. (2020) 9:3697. doi: 10.3390/jcm9113697 33217963 PMC7698715

[6] Hanna MG Parwani A Sirintrapun SJ . Whole slide imaging: technology and applications. Adv Anat Pathol. (2020) 27:251–9. doi: 10.1097/PAP.0000000000000273 32452840

[7] Hanna MG Reuter VE Hameed MR Tan LK Chiang S Sigel C . Whole slide imaging equivalency and efficiency study: experience at a large academic center. Mod Pathol. (2019) 32:916–28. doi: 10.1038/s41379-019-0205-0 30778169

[8] Schuettfort VM Pradere B Rink M Comperat E Shariat SF . Pathomics in urology. Curr Opin Urol. (2020) 30:823–31. doi: 10.1097/MOU.0000000000000813 32881725

[9] Buch VH Ahmed I Maruthappu M . Artificial intelligence in medicine: current trends and future possibilities. Br J Gen Pract. (2018) 68:143–4. doi: 10.3399/bjgp18X695213 PMC5819974 29472224

[10] Koteluk O Wartecki A Mazurek S Kołodziejczak I Mackiewicz A . How do machines learn? Artificial intelligence as a new era in medicine. J Pers Med. (2021) 11:32. doi: 10.3390/jpm11010032 33430240 PMC7825660

[11] Marletta S Eccher A Martelli FM Santonicco N Girolami I Scarpa A . Artificial intelligence-based algorithms for the diagnosis of prostate cancer: A systematic review. Am J Clin Pathol. (2024) 161:526–34. doi: 10.1093/ajcp/aqad182 38381582

[12] Marletta S L'Imperio V Eccher A Antonini P Santonicco N Girolami I . Artificial intelligence-based tools applied to pathological diagnosis of microbiological diseases. Pathol Res Pract. (2023) 243:154362. doi: 10.1016/j.prp.2023.154362 36758417

[13] Giulietti M Cecati M Sabanovic B Scire A Cimadamore A Santoni M . The role of artificial intelligence in the diagnosis and prognosis of renal cell tumors. Diagnostics (Basel). (2021) 11:206. doi: 10.3390/diagnostics11020206 33573278 PMC7912267

[14] Distante A Marandino L Bertolo R Ingels A Pavan N Pecoraro A . Artificial intelligence in renal cell carcinoma histopathology: current applications and future perspectives. Diagnostics (Basel). (2023) 13:2294. doi: 10.3390/diagnostics13132294 37443687 PMC10340141

[15] Dara S Tumma P eds. (2018). “Feature extraction by using deep learning: A survey”, in: 2018 Second International Conference on Electronics, Communication and Aerospace Technology (ICECA), 29-31 March 2018. United States: IEEE, Institute of Electrical and Electronics Engineers.

[16] Duan Y Lu J Zheng W Zhou J . Deep adversarial metric learning. IEEE Trans Image Process. (2020) 29:2037–51. doi: 10.1109/TIP.83 31670672

[17] Zhang Y Xi R Zeng L Towey D Bai R Higashita R . Structural priors guided network for the corneal endothelial cell segmentation. IEEE Trans Med Imaging. (2024) 43:309–20. doi: 10.1109/TMI.2023.3300656 37527299

[18] Khene ZE Kammerer-Jacquet SF Bigot P Rabilloud N Albiges L Margulis V . Clinical application of digital and computational pathology in renal cell carcinoma: A systematic review. Eur Urol Oncol. (2024) 7:401–11. doi: 10.1016/j.euo.2023.10.018 37925349

[19] Wessels F Kuntz S Krieghoff-Henning E Schmitt M Braun V Worst TS . Artificial intelligence to predict oncological outcome directly from hematoxylin and eosin-stained slides in urology. Minerva Urol Nephrol. (2022) 74:538–50. doi: 10.23736/S2724-6051.22.04758-9 35274903

[20] Page MJ McKenzie JE Bossuyt PM Boutron I Hoffmann TC Mulrow CD . The PRISMA 2020 statement: an updated guideline for reporting systematic reviews. Rev Panam Salud Publ. (2022) 46:89. doi: 10.1186/s13643-021-01626-4 PMC8008539 33781348

[21] Lipkova J Chen RJ Chen B Lu MY Barbieri M Shao D . Artificial intelligence for multimodal data integration in oncology. Cancer Cell. (2022) 40:1095–110. doi: 10.1016/j.ccell.2022.09.012 PMC10655164 36220072

[22] Lu MY Williamson DFK Chen TY Chen RJ Barbieri M Mahmood F . Data-efficient and weakly supervised computational pathology on whole-slide images. Nat BioMed Eng. (2021) 5:555–70. doi: 10.1038/s41551-020-00682-w PMC8711640 33649564

[23] Faust K Roohi A Leon AJ Leroux E Dent A Evans AJ . Unsupervised resolution of histomorphologic heterogeneity in renal cell carcinoma using a brain tumor-educated neural network. JCO Clin Cancer Inform. (2020) 4:811–21. doi: 10.1200/CCI.20.00035 PMC7529524 32946287

[24] Sarker IH . Deep learning: A comprehensive overview on techniques, taxonomy, applications and research directions. SN Comput Science. (2021) 2:420. doi: 10.1007/s42979-021-00815-1 PMC8372231 34426802

[25] Chen RJ Lu MY Wang J Williamson DFK Rodig SJ Lindeman NI . Pathomic fusion: an integrated framework for fusing histopathology and genomic features for cancer diagnosis and prognosis. IEEE Trans Med Imaging. (2022) 41:757–70. doi: 10.1109/TMI.2020.3021387 PMC10339462 32881682

[26] Xie Y Xu Z Zhang J Wang Z Ji S . Self-supervised learning of graph neural networks: A unified review. IEEE Trans Pattern Anal Mach Intell. (2023) 45:2412–29. doi: 10.1109/TPAMI.2022.3170559 PMC9902037 35476575

[27] Lee Y Park JH Oh S Shin K Sun J Jung M . Derivation of prognostic contextual histopathological features from whole-slide images of tumours via graph deep learning. Nat BioMed Eng. (2022). doi: 10.1038/s41551-022-00923-0 35982331

[28] Chen C Lu MY Williamson DFK Chen TY Schaumberg AJ Mahmood F . Fast and scalable search of whole-slide images via self-supervised deep learning. Nat BioMed Eng. (2022) 6:1420–34. doi: 10.1038/s41551-022-00929-8 PMC9792371 36217022

[29] DiPalma J Suriawinata AA Tafe LJ Torresani L Hassanpour S . Resolution-based distillation for efficient histology image classification. Artif Intell Med. (2021) 119:102136. doi: 10.1016/j.artmed.2021.102136 34531005 PMC8449014

[30] Chen RJ Lu MY Williamson DFK Chen TY Lipkova J Noor Z . Pan-cancer integrative histology-genomic analysis via multimodal deep learning. Cancer Cell. (2022) 40:865–78 e6. doi: 10.1016/j.ccell.2022.07.004 35944502 PMC10397370

[31] Shi M Li X Li M Si Y . Attention-based generative adversarial networks improve prognostic outcome prediction of cancer from multimodal data. Brief Bioinform. (2023) 24:bbad329. doi: 10.1093/bib/bbad329 37756592

[32] Schulz S Woerl AC Jungmann F Glasner C Stenzel P Strobl S . Multimodal deep learning for prognosis prediction in renal cancer. Front Oncol. (2021) 11:788740. doi: 10.3389/fonc.2021.788740 34900744 PMC8651560

[33] Fenstermaker M Tomlins SA Singh K Wiens J Morgan TM . Development and validation of a deep-learning model to assist with renal cell carcinoma histopathologic interpretation. Urology. (2020) 144:152–7. doi: 10.1016/j.urology.2020.05.094 32711010

[34] Abdeltawab H Khalifa F Ghazal M Cheng L Gondim D El-Baz A . A pyramidal deep learning pipeline for kidney whole-slide histology images classification. Sci Rep. (2021) 11:20189. doi: 10.1038/s41598-021-99735-6 34642404 PMC8511039

[35] Zhu M Ren B Richards R Suriawinata M Tomita N Hassanpour S . Development and evaluation of a deep neural network for histologic classification of renal cell carcinoma on biopsy and surgical resection slides. Sci Rep. (2021) 11:7080. doi: 10.1038/s41598-021-86540-4 33782535 PMC8007643

[36] Tabibu S Vinod PK Jawahar CV . Pan-Renal Cell Carcinoma classification and survival prediction from histopathology images using deep learning. Sci Rep. (2019) 9:10509. doi: 10.1038/s41598-019-46718-3 31324828 PMC6642160

[37] Marostica E Barber R Denize T Kohane IS Signoretti S Golden JA . Development of a histopathology informatics pipeline for classification and prediction of clinical outcomes in subtypes of renal cell carcinoma. Clin Cancer Res. (2021) 27:2868–78. doi: 10.1158/1078-0432.CCR-20-4119 33722896

[38] Ohe C Yoshida T Amin MB Uno R Atsumi N Yasukochi Y . Deep learning-based predictions of clear and eosinophilic phenotypes in clear cell renal cell carcinoma. Hum Pathol. (2023) 131:68–78. doi: 10.1016/j.humpath.2022.11.004 36372298

[39] Chen S Zhang N Jiang L Gao F Shao J Wang T . Clinical use of a machine learning histopathological image signature in diagnosis and survival prediction of clear cell renal cell carcinoma. Int J Cancer. (2021) 148:780–90. doi: 10.1002/ijc.v148.3 32895914

[40] Kruk M Kurek J Osowski S Koktysz R Swiderski B Markiewicz T . Ensemble of classifiers and wavelet transformation for improved recognition of Fuhrman grading in clear-cell renal carcinoma. Biocybernetics Biomed Engineering. (2017) 37:357–64. doi: 10.1016/j.bbe.2017.04.005

[41] Tian K Rubadue CA Lin DI Veta M Pyle ME Irshad H . Automated clear cell renal carcinoma grade classification with prognostic significance. PloS One. (2019) 14:e0222641. doi: 10.1371/journal.pone.0222641 31581201 PMC6776313

[42] Holdbrook DA Singh M Choudhury Y Kalaw EM Koh V Tan HS . Automated renal cancer grading using nuclear pleomorphic patterns. JCO Clin Cancer Inform. (2018) 2:1–12. doi: 10.1200/CCI.17.00100 30652593

[43] Chanchal AK Lal S Kumar R Kwak JT Kini J . A novel dataset and efficient deep learning framework for automated grading of renal cell carcinoma from kidney histopathology images. Sci Rep. (2023) 13:5728. doi: 10.1038/s41598-023-31275-7 37029115 PMC10082027

[44] Khoshdeli M Borowsky A Parvin B . Deep learning models differentiate tumor grades from H&E stained histology sections. Annu Int Conf IEEE Eng Med Biol Soc. (2018) 2018:620–3. doi: 10.1109/EMBC.2018.8512357 30440473

[45] Udager AM Mehra R . Morphologic, molecular, and taxonomic evolution of renal cell carcinoma: A conceptual perspective with emphasis on updates to the 2016 world health organization classification. Arch Pathol Lab Med. (2016) 140:1026–37. doi: 10.5858/arpa.2016-0218-RA 27684973

[46] Chen Q Kuai Y Wang S Zhu X Wang H Liu W . Deep learning-based classification of epithelial-mesenchymal transition for predicting response to therapy in clear cell renal cell carcinoma. Front Oncol. (2021) 11:782515. doi: 10.3389/fonc.2021.782515 35141144 PMC8819137

[47] Acosta PH Panwar V Jarmale V Christie A Jasti J Margulis V . Intratumoral resolution of driver gene mutation heterogeneity in renal cancer using deep learning. Cancer Res. (2022) 82:2792–806. doi: 10.1158/0008-5472.CAN-21-2318 PMC9373732 35654752

[48] Cheng J Han Z Mehra R Shao W Cheng M Feng Q . Computational analysis of pathological images enables a better diagnosis of TFE3 Xp11. 2 translocation Renal Cell carcinoma Nat Commun. (2020) 11:1778. doi: 10.1038/s41467-020-15671-5 32286325 PMC7156652

[49] Wei JH Feng ZH Cao Y Zhao HW Chen ZH Liao B . Predictive value of single-nucleotide polymorphism signature for recurrence in localised renal cell carcinoma: a retrospective analysis and multicentre validation study. Lancet Oncol. (2019) 20:591–600. doi: 10.1016/S1470-2045(18)30932-X 30880070

[50] Wessels F Schmitt M Krieghoff-Henning E Kather JN Nientiedt M Kriegmair MC . Deep learning can predict survival directly from histology in clear cell renal cell carcinoma. PloS One. (2022) 17:e0272656. doi: 10.1371/journal.pone.0272656 35976907 PMC9385058

[51] Gui CP Chen YH Zhao HW Cao JZ Liu TJ Xiong SW . Multimodal recurrence scoring system for prediction of clear cell renal cell carcinoma outcome: a discovery and validation study. Lancet Digit Health. (2023) 5:e515–e24. doi: 10.1016/S2589-7500(23)00095-X 37393162

[52] Cheng J Zhang J Han Y Wang X Ye X Meng Y . Integrative analysis of histopathological images and genomic data predicts clear cell renal cell carcinoma prognosis. Cancer Res. (2017) 77:e91–e100. doi: 10.1158/0008-5472.CAN-17-0313 29092949 PMC7262576

[53] Cheng J Mo X Wang X Parwani A Feng Q Huang K . Identification of topological features in renal tumor microenvironment associated with patient survival. Bioinformatics. (2018) 34:1024–30. doi: 10.1093/bioinformatics/btx723 PMC7263397 29136101

[54] Chen S Jiang L Gao F Zhang E Wang T Zhang N . Machine learning-based pathomics signature could act as a novel prognostic marker for patients with clear cell renal cell carcinoma. Br J Cancer. (2022) 126:771–7. doi: 10.1038/s41416-021-01640-2 PMC8888584 34824449

[55] Bankhead P Loughrey MB Fernández JA Dombrowski Y McArt DG Dunne PD . QuPath: Open source software for digital pathology image analysis. Sci Rep. (2017) 7:16878. doi: 10.1038/s41598-017-17204-5 29203879 PMC5715110

[56] Kalra S Tizhoosh HR Shah S Choi C Damaskinos S Safarpoor A . Pan-cancer diagnostic consensus through searching archival histopathology images using artificial intelligence. NPJ Digit Med. (2020) 3:31. doi: 10.1038/s41746-020-0238-2 32195366 PMC7064517

[57] Khodabakhshi Z Amini M Mostafaei S Haddadi Avval A Nazari M Oveisi M . Overall survival prediction in renal cell carcinoma patients using computed tomography radiomic and clinical information. J Digital Imaging. (2021) 34:1086–98. doi: 10.1007/s10278-021-00500-y PMC8554934 34382117

[58] Vanguri RS Luo J Aukerman AT Egger JV Fong CJ Horvat N . Multimodal integration of radiology, pathology and genomics for prediction of response to PD-(L)1 blockade in patients with non-small cell lung cancer. Nat Cancer. (2022) 3:1151–64. doi: 10.1038/s43018-022-00416-8 PMC9586871 36038778

[59] Miao J Thongprayoon C Garcia Valencia OA Krisanapan P Sheikh MS Davis PW . Performance of chatGPT on nephrology test questions. Clin J Am Soc Nephrol. (2024) 19(1):35–43. doi: 10.2215/CJN.0000000000000330 PMC10843340 37851468

