# Supplementary material for: Digital pathology and artificial intelligence in renal cell carcinoma focusing on feature extraction: a literature review
Source: Front Oncol. 2025 Jan 24;15:1516264. doi: 10.3389/fonc.2025.1516264 (PMC11802434; doi:10.3389/fonc.2025.1516264)
Supplement: Supplementary file 1 [file Table1.docx]

Supplementary material 1 The results of the analyses of the authors

| Value | Frequency | Percentage |
| --- | --- | --- |
| Lu, Ming Y. | 4 | 1.7094 |
| Mahmood, Faisal | 4 | 1.7094 |
| Williamson, Drew F. K. | 4 | 1.7094 |
| Chen, Richard J. | 3 | 1.2821 |
| Chen, Tiffany Y. | 3 | 1.2821 |
| Cheng, Jun | 3 | 1.2821 |
| Cheng, Liang | 3 | 1.2821 |
| Feng, Qianjin | 3 | 1.2821 |
| Huang, Kun | 3 | 1.2821 |
| Chen, Siteng | 2 | 0.8547 |
| Gao, Feng | 2 | 0.8547 |
| Han, Zhi | 2 | 0.8547 |
| Hassanpour, Saeed | 2 | 0.8547 |
| Jiang, Liren | 2 | 0.8547 |
| Parwani, Anil | 2 | 0.8547 |
| Wang, Tao | 2 | 0.8547 |
| Wang, Xiang | 2 | 0.8547 |
| Wang, Xusheng | 2 | 0.8547 |
| Zhang, Encheng | 2 | 0.8547 |
| Zhang, Jie | 2 | 0.8547 |
| Zhang, Ning | 2 | 0.8547 |
| Zheng, Junhua | 2 | 0.8547 |
