# Supplementary material for: Digital pathology and artificial intelligence in renal cell carcinoma focusing on feature extraction: a literature review
Source: Front Oncol. 2025 Jan 24;15:1516264. doi: 10.3389/fonc.2025.1516264 (PMC11802434; doi:10.3389/fonc.2025.1516264)
Supplement: Supplementary file 2 [file Table2.docx]

Supplementary material 2 The results of the analyses of the institutions

| Value | Frequency | Percentage |
| --- | --- | --- |
| Harvard Med Sch, Brigham & Womens Hosp, Dept Pathol | 4 | 2.8369 |
| Shanghai Jiao Tong Univ, Sch Med, Shanghai Gen Hosp | 4 | 2.8369 |
| Harvard Med Sch, Dept Biomed Informat, Boston | 3 | 2.1277 |
| Indiana Univ Sch Med, Dept Med, Indianapolis | 3 | 2.1277 |
| Broad Inst Harvard & MIT, Canc Program, Cambridge | 2 | 1.4184 |
| Dartmouth Coll, Dept Comp Sci, Hanover | 2 | 1.4184 |
| Dartmouth Hitchcock Med Ctr, Dept Pathol & Lab Med, Lebanon | 2 | 1.4184 |
| Geisel Sch Med Dartmouth, Dept Biomed Data Sci, Hanover | 2 | 1.4184 |
| Geisel Sch Med Dartmouth, Dept Epidemiol, Hanover | 2 | 1.4184 |
| Harvard Univ, Harvard Data Sci Initiat, Cambridge | 2 | 1.4184 |
| Heidelberg Univ, Univ Med Ctr Mannheim, Med Fac Mannheim | 2 | 1.4184 |
| Indiana Univ Sch Med, Dept Pathol & Lab Med, Indianapolis | 2 | 1.4184 |
| MIT, Dept Elect Engn & Comp Sci, Cambridge | 2 | 1.4184 |
| Ohio State Univ, Dept Biomed Informat, Columbus | 2 | 1.4184 |
| Ohio State Univ, Dept Pathol, Columbus | 2 | 1.4184 |
| Shanghai Jiao Tong Univ, Sch Med, Ruijin Hosp | 2 | 1.4184 |
| Southern Med Univ, Sch Biomed Engn, Guangdong Prov Key Lab Med Image Proc | 2 | 1.4184 |
