# Supplementary material for: Digital pathology and artificial intelligence in renal cell carcinoma focusing on feature extraction: a literature review
Source: Front Oncol. 2025 Jan 24;15:1516264. doi: 10.3389/fonc.2025.1516264 (PMC11802434; doi:10.3389/fonc.2025.1516264)
Supplement: Supplementary file 3 [file Table3.docx]

Supplementary material 3 The results of the analyses of the citations

| Value | Frequency | Percentage |
| --- | --- | --- |
| Coudray N, 2018, NAT MED, V24, P1559, DOI 10.1038/s41591-018-0177-5 | 14 | 1.3258 |
| Creighton CJ, 2013, NATURE, V499, P43, DOI 10.1038/nature12222 | 10 | 0.947 |
| Campanella G, 2019, NAT MED, V25, P1301, DOI 10.1038/s41591-019-0508-1 | 9 | 0.8523 |
| Yu KH, 2016, NAT COMMUN, V7, DOI 10.1038/ncomms12474 | 9 | 0.8523 |
| Esteva A, 2017, NATURE, V542, P115, DOI 10.1038/nature21056 | 7 | 0.6629 |
| Kather JN, 2019, NAT MED, V25, P1054, DOI 10.1038/s41591-019-0462-y | 7 | 0.6629 |
| Ricketts CJ, 2018, CELL REP, V23, P313, DOI [10.1016/j.celrep.2018.03.075, 10.1016/j.celrep.2018.06.032] | 7 | 0.6629 |
| Beck AH, 2011, SCI TRANSL MED, V3, DOI 10.1126/scitranslmed.3002564 | 6 | 0.5682 |
| Bejnordi BE, 2017, JAMA-J AM MED ASSOC, V318, P2199, DOI 10.1001/jama.2017.14585 | 6 | 0.5682 |
| Fu Y, 2020, NAT CANCER, V1, P800, DOI 10.1038/s43018-020-0085-8 | 5 | 0.4735 |
| He KM, 2016, PROC CVPR IEEE, P770, DOI 10.1109/CVPR.2016.90 | 5 | 0.4735 |
| Kather JN, 2020, NAT CANCER, V1, P789, DOI 10.1038/s43018-020-0087-6 | 5 | 0.4735 |
| Linehan WM, 2016, NEW ENGL J MED, V374, P135, DOI 10.1056/NEJMoa1505917 | 5 | 0.4735 |
| Mobadersany P, 2018, P NATL ACAD SCI USA, V115, pE2970, DOI 10.1073/pnas.1717139115 | 5 | 0.4735 |
| Tabibu S, 2019, SCI REP-UK, V9, DOI 10.1038/s41598-019-46718-3 | 5 | 0.4735 |
| Chen RJ, 2022, IEEE T MED IMAGING, V41, P757, DOI [10.1109/TMI.2020.3021387, 10.1109/TITS.2020.3030218] | 4 | 0.3788 |
| Courtiol P, 2019, NAT MED, V25, P1519, DOI 10.1038/s41591-019-0583-3 | 4 | 0.3788 |
| Delahunt B, 2013, AM J SURG PATHOL, V37, P1490, DOI 10.1097/PAS.0b013e318299f0fb | 4 | 0.3788 |
| Ilse M, 2018, PR MACH LEARN RES, V80 | 4 | 0.3788 |
| Kather JN, 2019, PLOS MED, V16, DOI 10.1371/journal.pmed.1002730 | 4 | 0.3788 |
| Kovacs G, 1997, J PATHOL, V183, P131, DOI 10.1002/(SICI)1096-9896(199710)183:2<131::AID-PATH931>3.0.CO;2-G | 4 | 0.3788 |
| Phoulady HA, 2016, PROC SPIE, V9791, DOI 10.1117/12.2216632 | 4 | 0.3788 |
| Amin MB, 2002, AM J SURG PATHOL, V26, P281, DOI 10.1097/00000478-200203000-00001 | 3 | 0.2841 |
| Bankhead P, 2017, SCI REP-UK, V7, DOI 10.1038/s41598-017-17204-5 | 3 | 0.2841 |
| Bera K, 2019, NAT REV CLIN ONCOL, V16, P703, DOI 10.1038/s41571-019-0252-y | 3 | 0.2841 |
| Cerami E, 2012, CANCER DISCOV, V2, P401, DOI 10.1158/2159-8290.CD-12-0095 | 3 | 0.2841 |
| Cruz-Roa A, 2017, SCI REP-UK, V7, DOI 10.1038/srep46450 | 3 | 0.2841 |
| Davis CF, 2014, CANCER CELL, V26, P319, DOI 10.1016/j.ccr.2014.07.014 | 3 | 0.2841 |
| Friedman J, 2010, J STAT SOFTW, V33, P1, DOI 10.18637/jss.v033.i01 | 3 | 0.2841 |
| Gutman DA, 2013, J AM MED INFORM ASSN, V20, P1091, DOI 10.1136/amiajnl-2012-001469 | 3 | 0.2841 |
| Hipp Jason, 2011, J Pathol Inform, V2, P25, DOI 10.4103/2153-3539.82050 | 3 | 0.2841 |
| Kingma D. P., 2014, arXiv, DOI DOI 10.48550/ARXIV.1412.6980 | 3 | 0.2841 |
| Ljungberg B, 2019, EUR UROL, V75, P799, DOI 10.1016/j.eururo.2019.02.011 | 3 | 0.2841 |
| Marostica E, 2021, CLIN CANCER RES, V27, P2868, DOI 10.1158/1078-0432.CCR-20-4119 | 3 | 0.2841 |
| Muglia Valdair F., 2015, Radiol Bras, V48, P166, DOI 10.1590/0100-3984.2013.1927 | 3 | 0.2841 |
| Russakovsky O, 2015, INT J COMPUT VISION, V115, P211, DOI 10.1007/s11263-015-0816-y | 3 | 0.2841 |
| Schapiro D, 2017, NAT METHODS, V14, P873, DOI [10.1038/NMETH.4391, 10.1038/nmeth.4391] | 3 | 0.2841 |
| Siegel RL, 2021, CA-CANCER J CLIN, V71, P7, DOI [10.3322/caac.21387, 10.3322/caac.21654, 10.3322/caac.21551, 10.3322/caac.21254, 10.3322/caac.21601, 10.3322/caac.20006, 10.3322/caac.21332, 10.3322/caac.20073] | 3 | 0.2841 |
| Simonyan K, 2015, Arxiv, DOI [arXiv:1409.1556, DOI 10.48550/ARXIV.1409.1556] | 3 | 0.2841 |
| Skrede OJ, 2020, LANCET, V395, P350, DOI 10.1016/S0140-6736(19)32998-8 | 3 | 0.2841 |
| Sundararajan M, 2017, PR MACH LEARN RES, V70 | 3 | 0.2841 |
| Vahadane A, 2016, IEEE T MED IMAGING, V35, P1962, DOI 10.1109/TMI.2016.2529665 | 3 | 0.2841 |
| Yuan YY, 2012, SCI TRANSL MED, V4, DOI 10.1126/scitranslmed.3004330 | 3 | 0.2841 |
| Abdelmoula WM, 2016, P NATL ACAD SCI USA, V113, P12244, DOI 10.1073/pnas.1510227113 | 2 | 0.1894 |
| AbdulJabbar K, 2020, NAT MED, V26, P1054, DOI 10.1038/s41591-020-0900-x | 2 | 0.1894 |
| Amin MB, 2017, CA-CANCER J CLIN, V67, P93, DOI 10.3322/caac.21388 | 2 | 0.1894 |
| Argani P, 2015, SEMIN DIAGN PATHOL, V32, P103, DOI 10.1053/j.semdp.2015.02.003 | 2 | 0.1894 |
| Baltrusaitis T, 2019, IEEE T PATTERN ANAL, V41, P423, DOI 10.1109/TPAMI.2018.2798607 | 2 | 0.1894 |
| Beer DG, 2002, NAT MED, V8, P816, DOI 10.1038/nm733 | 2 | 0.1894 |
| Bland JM, 2004, BRIT MED J, V328, P1073, DOI 10.1136/bmj.328.7447.1073 | 2 | 0.1894 |
| Braun DA, 2020, NAT MED, V26, P909, DOI 10.1038/s41591-020-0839-y | 2 | 0.1894 |
| Bulten W, 2020, LANCET ONCOL, V21, P233, DOI 10.1016/S1470-2045(19)30739-9 | 2 | 0.1894 |
| Cai Q, 2020, EBIOMEDICINE, V51, DOI 10.1016/j.ebiom.2019.10.052 | 2 | 0.1894 |
| Cancer Genome Atlas Research Network, 2013, Nat Genet, V45, P1113, DOI 10.1038/ng.2764 | 2 | 0.1894 |
| Cheerla A, 2019, BIOINFORMATICS, V35, pI446, DOI 10.1093/bioinformatics/btz342 | 2 | 0.1894 |
| Chen JM, 2015, SCI REP-UK, V5, DOI 10.1038/srep10690 | 2 | 0.1894 |
| Chen RJ, 2021, LECT NOTES COMPUT SC, V12908, P339, DOI 10.1007/978-3-030-87237-3_33 | 2 | 0.1894 |
| Chen ST, 2021, INT J CANCER, V148, P780, DOI 10.1002/ijc.33288 | 2 | 0.1894 |
| Cheng J, 2017, CANCER RES, V77, pE91, DOI 10.1158/0008-5472.CAN-17-0313 | 2 | 0.1894 |
| Cheng J, 2018, BIOINFORMATICS, V34, P1024, DOI 10.1093/bioinformatics/btx723 | 2 | 0.1894 |
| Cheville JC, 2003, AM J SURG PATHOL, V27, P612, DOI 10.1097/00000478-200305000-00005 | 2 | 0.1894 |
| DeCastro GJ, 2008, UROL CLIN N AM, V35, P581, DOI 10.1016/j.ucl.2008.07.005 | 2 | 0.1894 |
| Delahunt B, 2019, HISTOPATHOLOGY, V74, P4, DOI 10.1111/his.13735 | 2 | 0.1894 |
| Esteva A, 2019, NAT MED, V25, P24, DOI 10.1038/s41591-018-0316-z | 2 | 0.1894 |
| Faust K, 2020, JCO CLIN CANCER INFO, V4, P811, DOI 10.1200/CCI.20.00035 | 2 | 0.1894 |
| Gao JJ, 2013, SCI SIGNAL, V6, DOI 10.1126/scisignal.2004088 | 2 | 0.1894 |
| Goyal R, 2013, ARCH PATHOL LAB MED, V137, P467, DOI 10.5858/arpa.2012-0085-RA | 2 | 0.1894 |
| Graham S, 2019, MED IMAGE ANAL, V58, DOI 10.1016/j.media.2019.101563 | 2 | 0.1894 |
| Gutierrez Olivares Victor Manuel, 2019, Rev Esp Patol, V52, P214, DOI 10.1016/j.patol.2019.02.004 | 2 | 0.1894 |
| He B, 2020, NAT BIOMED ENG, V4, P827, DOI 10.1038/s41551-020-0578-x | 2 | 0.1894 |
| He K., 2015, PROC IEEE INT C COMP, P1026, DOI DOI 10.1109/ICCV.2015.123 | 2 | 0.1894 |
| Heagerty PJ, 2000, BIOMETRICS, V56, P337, DOI 10.1111/j.0006-341X.2000.00337.x | 2 | 0.1894 |
| Heng DYC, 2009, J CLIN ONCOL, V27, P5794, DOI 10.1200/JCO.2008.21.4809 | 2 | 0.1894 |
| Holdbrook DA, 2018, JCO CLIN CANCER INFO, V2, DOI 10.1200/CCI.17.00100 | 2 | 0.1894 |
| Hollon TC, 2020, NAT MED, V26, P52, DOI 10.1038/s41591-019-0715-9 | 2 | 0.1894 |
| Hong SK, 2011, BJU INT, V107, P409, DOI 10.1111/j.1464-410X.2010.09561.x | 2 | 0.1894 |
| Hsieh JJ, 2017, NAT REV DIS PRIMERS, V3, DOI 10.1038/nrdp.2017.9 | 2 | 0.1894 |
| Inamura K, 2005, ONCOGENE, V24, P7105, DOI 10.1038/sj.onc.1208858 | 2 | 0.1894 |
| Irshad Humayun, 2014, IEEE Rev Biomed Eng, V7, P97, DOI 10.1109/RBME.2013.2295804 | 2 | 0.1894 |
| Jackson HW, 2020, NATURE, V578, P615, DOI 10.1038/s41586-019-1876-x | 2 | 0.1894 |
| Jemal A, 2011, CA-CANCER J CLIN, V61, P134, DOI [10.3322/caac.20115, 10.3322/caac.21492, 10.3322/caac.20107] | 2 | 0.1894 |
| Kapur P, 2013, LANCET ONCOL, V14, P159, DOI 10.1016/S1470-2045(12)70584-3 | 2 | 0.1894 |
| Kickingereder P, 2019, LANCET ONCOL, V20, P728, DOI 10.1016/S1470-2045(19)30098-1 | 2 | 0.1894 |
| Kokhlikyan N, 2020, Arxiv, DOI [arXiv:2009.07896, 10.48550/arXiv.2009.07896, DOI 10.48550/ARXIV.2009.07896] | 2 | 0.1894 |
| Korbar Bruno, 2017, J Pathol Inform, V8, P30, DOI 10.4103/jpi.jpi_34_17 | 2 | 0.1894 |
| Kulkarni PM, 2020, CLIN CANCER RES, V26, P1126, DOI 10.1158/1078-0432.CCR-19-1495 | 2 | 0.1894 |
| Kumar N, 2017, IEEE T MED IMAGING, V36, P1550, DOI 10.1109/TMI.2017.2677499 | 2 | 0.1894 |
| Lang H, 2005, CANCER, V103, P625, DOI 10.1002/cncr.20812 | 2 | 0.1894 |
| Liu Y, 2017, COMMUN MATH BIOL NEU, DOI 10.1080/10408398.2017.1329704 | 2 | 0.1894 |
| Ljungberg B, 2015, EUR UROL, V67, P913, DOI 10.1016/j.eururo.2015.01.005 | 2 | 0.1894 |
| Louis DN, 2016, ACTA NEUROPATHOL, V131, P803, DOI 10.1007/s00401-016-1545-1 | 2 | 0.1894 |
| Lu MY, 2021, NAT BIOMED ENG, V5, P555, DOI 10.1038/s41551-020-00682-w | 2 | 0.1894 |
| Lu MY, 2021, NATURE, V594, P106, DOI 10.1038/s41586-021-03512-4 | 2 | 0.1894 |
| Mahmood F, 2020, IEEE T MED IMAGING, V39, P3257, DOI 10.1109/TMI.2019.2927182 | 2 | 0.1894 |
| Marusyk A, 2012, NAT REV CANCER, V12, P323, DOI 10.1038/nrc3261 | 2 | 0.1894 |
| Moch H, 2016, EUR UROL, V70, P93, DOI 10.1016/j.eururo.2016.02.029 | 2 | 0.1894 |
| Naylor P, 2019, IEEE T MED IMAGING, V38, P448, DOI 10.1109/TMI.2018.2865709 | 2 | 0.1894 |
| Niazi MKK, 2019, LANCET ONCOL, V20, pE253, DOI 10.1016/S1470-2045(19)30154-8 | 2 | 0.1894 |
| Paszke A, 2019, ADV NEUR IN, V32 | 2 | 0.1894 |
| Patard JJ, 2005, J CLIN ONCOL, V23, P2763, DOI 10.1200/JCO.2005.07.055 | 2 | 0.1894 |
| Prasad SR, 2006, RADIOGRAPHICS, V26, P1795, DOI 10.1148/rg.266065010 | 2 | 0.1894 |
| R Core Team, 2020, R: A Language and Environment for Statistical Computing | 2 | 0.1894 |
| Rusk N, 2016, NAT METHODS, V13, P35, DOI 10.1038/nmeth.3707 | 2 | 0.1894 |
| Saltz J, 2018, CELL REP, V23, P181, DOI 10.1016/j.celrep.2018.03.086 | 2 | 0.1894 |
| Samaratunga H, 2014, J KIDNEY CANCER VHL, V1, P26, DOI 10.15586/jkcvhl.2014.11 | 2 | 0.1894 |
| Schaumberg AJ, 2020, MODERN PATHOL, V33, P2169, DOI 10.1038/s41379-020-0540-1 | 2 | 0.1894 |
| Selvaraju RR, 2020, INT J COMPUT VISION, V128, P336, DOI [10.1109/ICCV.2017.74, 10.1007/s11263-019-01228-7] | 2 | 0.1894 |
| Shi JY, 2020, SENSORS-BASEL, V20, DOI 10.3390/s20174750 | 2 | 0.1894 |
| Simon N, 2011, J STAT SOFTW, V39, P1 | 2 | 0.1894 |
| Szegedy C, 2016, PROC CVPR IEEE, P2818, DOI 10.1109/CVPR.2016.308 | 2 | 0.1894 |
| Therneau T. M., 2015, PACKAGE SURVIVAL ANA, V2, P38 | 2 | 0.1894 |
| Thorsson V, 2019, IMMUNITY, V51, P411, DOI [10.1016/j.immuni.2019.08.004, 10.1016/j.immuni.2018.03.023] | 2 | 0.1894 |
| Tian K, 2019, PLOS ONE, V14, DOI 10.1371/journal.pone.0222641 | 2 | 0.1894 |
| Tomita N, 2019, JAMA NETW OPEN, V2, DOI 10.1001/jamanetworkopen.2019.14645 | 2 | 0.1894 |
| Travis WD, 2015, J THORAC ONCOL, V10, P1243, DOI 10.1097/JTO.0000000000000630 | 2 | 0.1894 |
| Wang C, 2013, J AM MED INFORM ASSN, V20, P680, DOI 10.1136/amiajnl-2012-001538 | 2 | 0.1894 |
| Wang SJ, 2019, MED IMAGE ANAL, V58, DOI 10.1016/j.media.2019.101549 | 2 | 0.1894 |
| Wei JW, 2019, SCI REP-UK, V9, DOI 10.1038/s41598-019-40041-7 | 2 | 0.1894 |
| Wei Jason W, 2019, J Pathol Inform, V10, P7, DOI 10.4103/jpi.jpi_87_18 | 2 | 0.1894 |
| Woerl AC, 2020, EUR UROL, V78, P256, DOI 10.1016/j.eururo.2020.04.023 | 2 | 0.1894 |
| Xu J, 2016, IEEE T MED IMAGING, V35, P119, DOI 10.1109/TMI.2015.2458702 | 2 | 0.1894 |
| Yang YL, 2011, BIOMED OPT EXPRESS, V2, P2160, DOI 10.1364/BOE.2.002160 | 2 | 0.1894 |
| Yeh Fang-Cheng, 2014, J Pathol Inform, V5, P23, DOI 10.4103/2153-3539.137726 | 2 | 0.1894 |
| Zhang XF, 2015, IEEE T MED IMAGING, V34, P496, DOI 10.1109/TMI.2014.2361481 | 2 | 0.1894 |
| Zhang XF, 2015, MED IMAGE ANAL, V26, P306, DOI 10.1016/j.media.2015.10.005 | 2 | 0.1894 |
| Zhao Y, 2020, PROC CVPR IEEE, P4836, DOI 10.1109/CVPR42600.2020.00489 | 2 | 0.1894 |
| Zhou YN, 2019, IEEE INT CONF COMP V, P388, DOI 10.1109/ICCVW.2019.00050 | 2 | 0.1894 |
| Zhu MD, 2021, SCI REP-UK, V11, DOI 10.1038/s41598-021-86540-4 | 2 | 0.1894 |
| Znaor A, 2015, EUR UROL, V67, P519, DOI 10.1016/j.eururo.2014.10.002 | 2 | 0.1894 |
