# Supplementary material for: Digital pathology and artificial intelligence in renal cell carcinoma focusing on feature extraction: a literature review
Source: Front Oncol. 2025 Jan 24;15:1516264. doi: 10.3389/fonc.2025.1516264 (PMC11802434; doi:10.3389/fonc.2025.1516264)
Supplement: Supplementary file 4 [file Table4.docx]

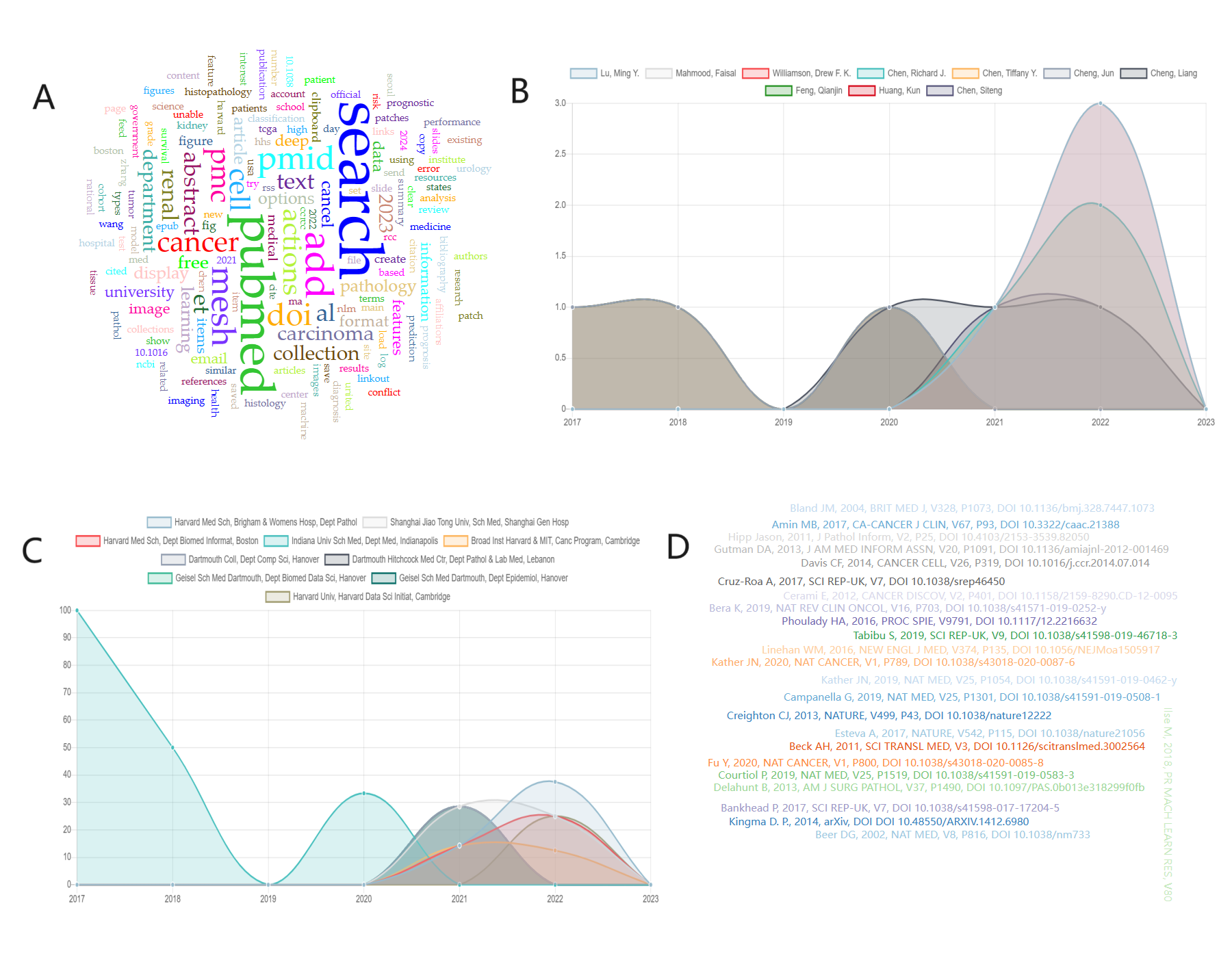


**Supplementary material 4** Full text analysis of Voyant and SATI

**A.** Keyword cloud on Voyant

**B.** Time series of author information frequency

**C.** Time series of rate of organizations

**D.** Citations nephogram

Rate = frequency/total of the year 100. The frequency refers to the number of times the entry appears in a certain year; the rate is the percentage of occurrences of the entry in a certain year compared to the total number of entries.
